# MetaOmixTools: A User-Friendly Web Suite for Meta-analysis of Ranked Features and Functional Enrichment

**DOI:** 10.34133/csbj.0157

**Published:** 2026-06-30

**Authors:** Rubén Grillo-Risco, Maksym Kupchyk Tiurin, Carla Perpiñá-Clérigues, Francisco J. Cordero Felipe, Samuel Lozano Juárez, María Iglesia-Vayá, Francisco García-García

**Affiliations:** ^1^ Computational Biomedicine Laboratory, Principe Felipe Research Centre (CIPF), Valencia 46012, Spain.; ^2^ Molecular, Cellular and Genomic Biomedicine Research Group, Instituto de Investigación Sanitaria La Fe (IIS La Fe), Valencia, Spain.; ^3^ Tuberculosis Genomics Unit, Instituto de Biomedicina de Valencia (IBV), CSIC, Valencia, Spain.; ^4^ Cardiovascular Proteomics Laboratory, Centro Nacional de Investigaciones Cardiovasculares Carlos III (CNIC), Madrid 28029, Spain.; ^5^ Escuela de Doctorado, Universidad Autónoma de Madrid, Madrid, Spain.; ^6^ Joint Unit in Biomedical Imaging and Artificial Intelligence FISABIO-CIPF, Foundation for the Promotion of Health and Biomedical Research of Valencia Region, Valencia, Spain.

## Abstract

The growing number of omics datasets in public repositories provides an opportunity to enhance data reusability through data integration; however, complex statistical barriers often hinder the effective combination of independent studies. To address this problem, we present MetaOmixTools, an interactive web-based suite that streamlines the meta-analysis of ranked feature lists and functional enrichment profiles. The platform integrates 2 primary modules—MetaRank and MetaEnrich—within a code-free environment. MetaRank generates robust consensus rankings from multiple lists by implementing weighted (e.g., rank product) and unweighted (e.g., robust rank aggregation) strategies, while MetaEnrich performs functional meta-analyses by combining probability values from individual overrepresentation analyses using established statistical techniques. Using case studies, we established consensus rankings for acute spinal cord injury across heterogeneous platforms, identifying conserved inflammatory marker genes in the up-regulated gene list (e.g., *Slpi*, *Ccl2*, and *Msr1*) and synaptic loss genes in the down-regulated gene list (e.g., *Kcna2*, *Dao*, and *Ppp1r1b*), and also characterized inverse functional intersections between melanoma brain metastasis and neurodegenerative diseases. By providing intuitive, real-time visualization and reproducible workflows, MetaOmixTools empowers the research community to extract consistent biological insights from multistudy data. We have made MetaOmixTools freely available at https://bioinfo.cipf.es/metaomixtools/.

## Highlights

• MetaOmixTools is an interactive, web-based platform that streamlines the meta-analysis of ranked feature lists and functional enrichment profiles, including 2 powerful analysis modules: MetaRank and MetaEnrich.
• MetaRank generates robust consensus rankings across studies using both weighted (e.g., rank product) and unweighted (e.g., robust rank aggregation) methods.
• MetaEnrich performs functional meta-analysis by integrating *P* values from independent overrepresentation analyses using established statistical approaches.
• The platform offers intuitive visualization and reproducible workflows, enabling consistent extraction of biological insights from multistudy omics data.

## Graphical Abstract

**Figure:**
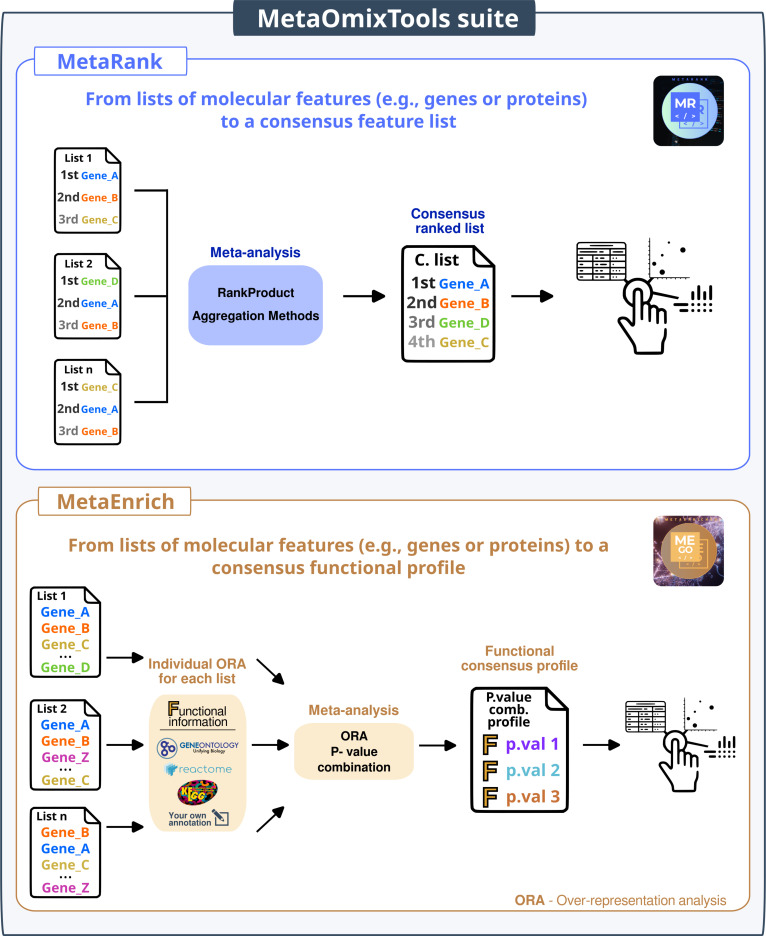

## Background

High-throughput technologies have generated a rapidly increasing number of omics datasets for humans and model organisms. Public repositories such as Gene Expression Omnibus (GEO) and ArrayExpress contain thousands of studies that address similar biological questions [1,2]. Combining results across multiple datasets substantially increases reproducibility and statistical power, particularly in situations where individual studies remain small or have been conducted under heterogeneous experimental conditions [3–5]. Two meta-analysis strategies hold special relevance for this purpose: (a) integrating ranked feature lists to produce a single consensus ranking and (b) combining *P* values from functional enrichment analyses to create a consensus functional profile.

Rank aggregation methods enable researchers to identify those genes, proteins, or other molecular entities consistently ranking at the top across lists from multiple sources; for example, these methods integrate differential gene expression analyses or prioritize genes from independent genomic variation studies using pathogenicity indicators [6–8]. This approach employs diverse methodologies, with the choice of method depending on the nature of the input data. The availability of quantitative scores allows the application of weighted strategies (e.g., log fold changes [logFC] or *P* values), whereas unweighted approaches apply when only the rank order represents a known quality.

While various R packages exist in the literature, RankProd and the robust rank aggregation (RRA) algorithm remain the most widely adopted tools for weighted and unweighted approaches, respectively. Their widespread adoption, with more than 2,000 citations to date, provides evidence of the reliability and robustness of these methodologies [9–11].

Meta-analysis for functional enrichment has been applied since the expansion of omics data in the early 2010s to identify shared biological functions across multiple feature lists [12–15]; meta-analysis in this context is typically based on *P*-value combination methods. The metap R package implements several standard statistical methods for this purpose (e.g., Fisher’s, Stouffer’s, Tippett’s, and Wilkinson’s methods) [16–19] and is actively maintained on The Comprehensive R Archive Network (https://cran.r-project.org). The thousands of monthly downloads reflect the continued utility of this package in statistical and bioinformatics workflows.

Although R packages contain these methodologies, a reliance on programming remains an important barrier for many researchers. Web-based tools have been developed to make these approaches more accessible. However, existing platforms often present limitations such as a restricted range of meta-analysis methods, inflexible input formats, or obsolescence due to lack of maintenance [12,20,21]. Consequently, there is a need for intuitive, well-maintained platforms that facilitate access to meta-analysis methods without compromising statistical rigor and providing flexible statistical strategies.

To bridge this gap, we introduce MetaOmixTools as an open-access, registration-free web application that integrates the MetaRank and MetaEnrich frameworks. The platform offers an interactive interface that guides users through the entire workflow, from data upload to result interpretation, generating customizable, real-time visualizations. Rather than introducing new statistical models, MetaOmixTools acts as a wrapper for existing statistical methods. By eliminating the need for coding expertise, MetaOmixTools democratizes ranking-based and *P*-value-based meta-analysis, making sophisticated data integration accessible to bioinformaticians and experimental biologists alike.

## Methods

**Fig. 1.**
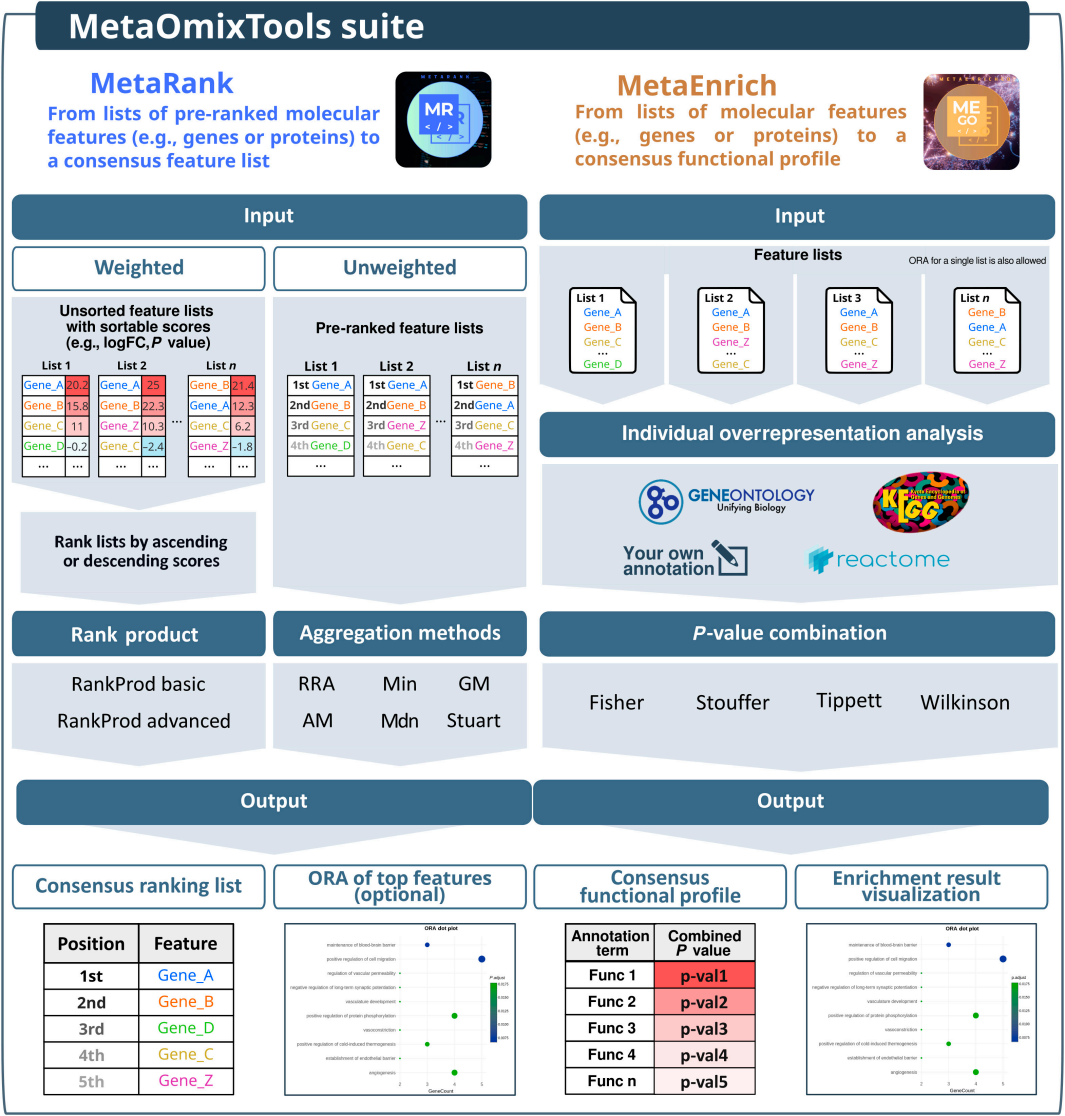
Overview of the MetaOmixTools suite workflow. The MetaRank module (left) integrates multiple lists of molecular features, provided either as unsorted lists with associated scores (weighted input) or as pre-ranked lists (unweighted input). In weighted mode, features are ordered by ascending or descending scores and combined using rank-based meta-analysis methods, including rank product (RP) (basic and advanced). The unweighted mode applies aggregation strategies such as robust rank aggregation (RRA), minimum (Min), geometric mean (GM), arithmetic mean (AM), median (Mdn), and Stuart’s method. The main output is a consensus ranking list, with optional overrepresentation analysis (ORA) applied to top-ranked features. The MetaEnrich module (right) performs functional enrichment meta-analysis from multiple feature lists. ORA is first carried out individually for each list using annotation databases (Gene Ontology, Kyoto Encyclopedia of Genes and Genomes, and Reactome) or user-defined annotations, and the resulting *P* values are then combined across lists using methods (Fisher’s, Stouffer’s, Tippett’s, or Wilkinson’s) to generate a consensus functional profile. As a secondary option, individual ORAs can also be performed on a single feature list without the meta-ranking step. All outputs include interactive visualizations and downloadable tables and plots.

The application structure is modular, comprising 2 primary analytical modules—MetaRank and MetaEnrich—implemented as independent components yet integrated into a unified user interface (Fig. 1). For further details about the statistical methods, workflow, or background pipeline, an extensive manual can be found in the User Guide module of the web platform. Additionally, the complete source code is openly available at https://github.com/emekate00/metaomixtools.

### MetaRank module

The MetaRank module, designed to obtain a robust consensus ranking from multiple feature lists (e.g., genes or proteins) to identify consistently relevant elements across studies, implements 2 distinct statistical approaches depending on the nature of the input data (Fig. 1, left panel). Weighted lists containing quantitative metrics (e.g., fold changes or *P* values) implement the rank product method via the RankProd package [10]. This nonparametric approach calculates the geometric mean of ranks for each feature across all replicates and assesses significance against a null distribution generated by permutation, yielding a percentage of false prediction score equivalent to the false discovery rate (FDR). For unweighted pre-ranked lists, the module integrates the RRA algorithm using the RobustRankAggreg package [11]. RRA uses a probabilistic model based on order statistics to detect features ranked consistently better than expected under a random null model. Alternative aggregation methods, such as geometric mean or median rank, are also available for specific research needs. Finally, to facilitate biological interpretation, the module allows users to perform an optional overrepresentation analysis (ORA) on the top-ranked features of the generated consensus list.

### MetaEnrich module

The MetaEnrich module performs functional meta-analysis by combining enrichment results across multiple feature lists. The workflow consists of 2 sequential statistical steps (Fig. 1, right panel). First, an ORA is executed independently for each input feature list using the hypergeometric test implemented in the enricher function of the clusterProfiler package [22]. Features are mapped to biological terms using internal local databases (Gene Ontology [GO], Kyoto Encyclopedia of Genes and Genomes [KEGG], or Reactome) for *Homo sapiens*, *Mus musculus*, and *Rattus norvegicus* or via custom user-provided annotations. Second, the module statistically combines the *P* values obtained for identical functional terms across different lists to derive a global consensus significance. The metap package [23] was used to implement several classic *P*-value combination methods, enabling users to tailor the analysis to their specific biological hypotheses. These options include Fisher’s (sum of logs), Stouffer’s (sum of *z*-scores), Tippett’s (minimum *P* value), and Wilkinson’s (maximization of *P* values) methods [16–19]. The final consensus *P* values are adjusted for multiple hypothesis testing using the Benjamini–Hochberg method [24]. Beyond multilist meta-analysis, the module offers the flexibility to analyze a single feature list, serving as a conventional ORA tool without integration.

### Implementation and user interface

**Fig. 2.**
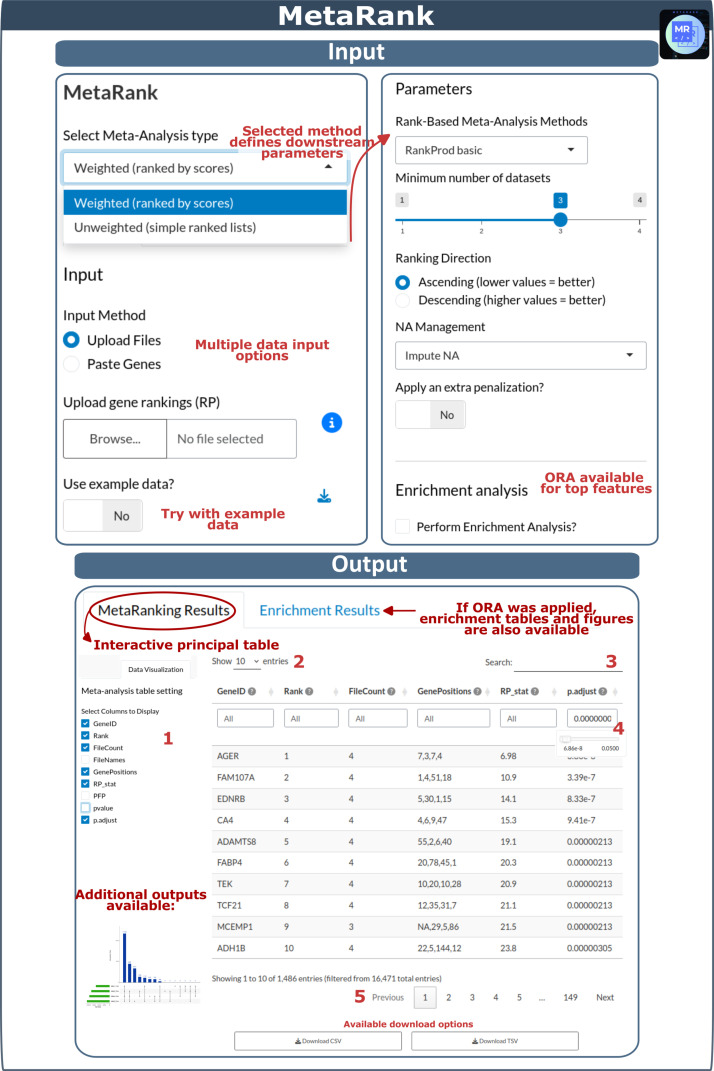
MetaRank interface. Input and output options of the MetaRank module and data visualization panel are shown. Multiple data input options are supported, including file upload, manual gene input, and execution with example data. The selected meta-analysis strategy automatically defines downstream parameters. Users can configure the minimum number of datasets required, the ranking direction based on the input metric, and strategies for missing value management. Overrepresentation analysis (ORA) can be optionally applied to top-ranked features. Results are displayed as an interactive meta-ranking table, with additional secondary outputs available. Numbers indicate standard DT table options for searching, filtering, pagination, and column-specific queries. All result tables and plots can be downloaded for further analysis or reporting. Red numbers indicate options for data customization and exploration, representing column visibility settings (1), the number of rows displayed per page (2), the global search bar (3), column-specific filters (4), and pagination controls (5). If an ORA was applied during configuration, enrichment tables and figures become available in the secondary tab, allowing users to inspect additional outputs such as summary plots. All results can be exported locally using the available download options located at the bottom of the interface.

MetaOmixTools was developed using R (v4.3.3) and deployed as an interactive web application using the Shiny framework (v1.10), utilizing HTML and CSS for enhanced front-end interactivity and responsiveness (Fig. 2). The application architecture is modular, with each module encapsulating its own user interface logic, back-end R scripts, test datasets, and graphical resources while sharing core utility functions for data processing.

**Fig. 3.**
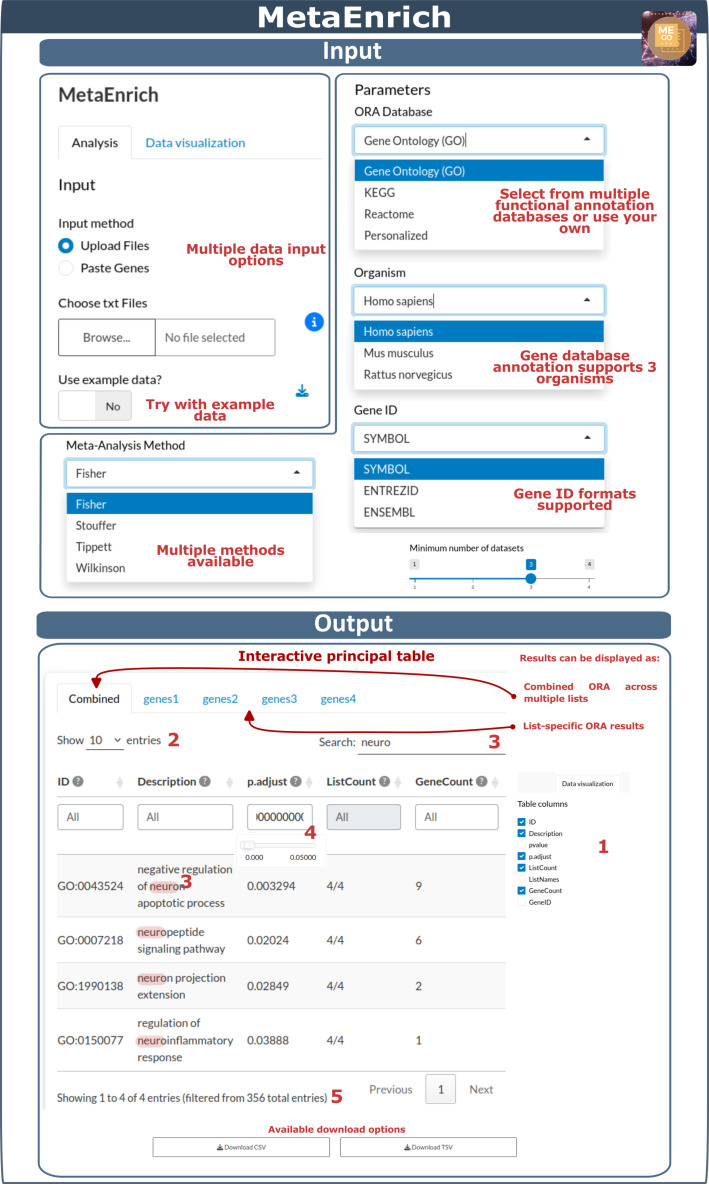
MetaEnrich interface. Input and output options of the MetaEnrich module are shown. Multiple data input options are supported, including file upload, manual gene input, and execution with example data. Users can select from multiple functional annotation databases or provide their own custom annotations. The platform supports functional analysis across 3 model organisms and accommodates several standard gene identifier formats. Multiple statistical methods are available for *P*-value combination, alongside a sliding threshold to filter terms based on their minimum frequency of appearance across the independent lists. Functional enrichment meta-analysis results are displayed within an interactive principal table, allowing users to toggle between the combined analysis across multiple lists and individual list-specific enrichment results. Red numbers indicate options for data customization and exploration, representing column visibility settings (1), the number of rows displayed per page (2), the global search bar (3), column-specific filters (4), and pagination controls (5). All results can be exported locally using the available download options located at the bottom of the interface.

To facilitate ease of use, both modules share standardized input and output mechanisms. Data can be introduced via multiple file uploads, by direct pasting of feature lists, or by loading built-in example datasets. Similarly, outputs are harmonized across the platform. Tabular results are displayed using interactive DT tables [25] with filtering, sorting, and export options (Figs. 2 and 3).

The MetaRank interface allows users to easily toggle between weighted and unweighted meta-analysis modes; crucially, the interface provides specific mechanisms for handling missing identifiers across heterogeneous datasets. Users can choose to impute missing ranks with the median rank of the list, ignore incomplete cases, or penalize missing features by assigning them the worst possible rank, ensuring that the final consensus reflects global consistency across studies (Fig. 2). Users can find a complete guide, benchmarking, comparison, and a thorough explanation of these strategies in the online User Guide.

The MetaEnrich interface allows users to select the *P*-value combination method. The incorporation of local, lightweight annotation databases across major model organisms is a critical feature of this design, ensuring full functionality and speed without reliance on external application programming interfaces during execution. Furthermore, users can also upload custom files. Additionally, the interface includes filtering options, such as defining the minimum appearance frequency of a feature across lists to be considered in the combination, allowing for stricter consensus definitions (Fig. 3).

**Fig. 4.**
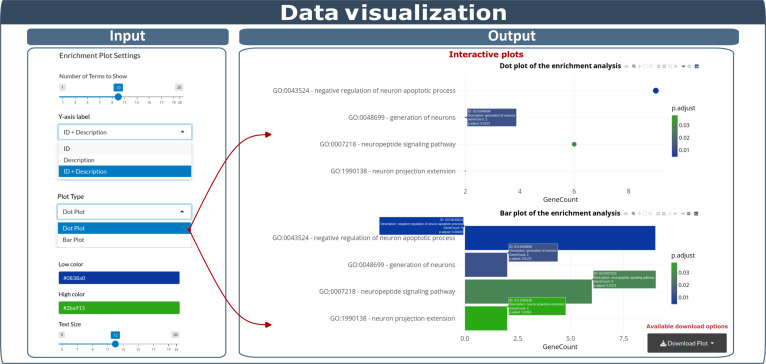
Data visualization. Overrepresentation analysis (ORA) results for both modules allow interactive visualization using dot or bar plots, with customizable display parameters (e.g., number of terms, axis labels, color gradients, and text size). Different plot configurations are available, including the number of terms, *y*-axis labels, plot type, text size, and custom hexadecimal color gradients. The right panel displays the resulting interactive plots generated by the suite. Users can hover over specific elements to display descriptive tooltips or export the figures via the download button at the bottom.

The visualization engine represents a core component of the user experience (Fig. 4). The Enrichment Analysis Plot panel renders results from both modules using dynamic graphics implemented with ggplot2 [26] and plotly [27]. Users can visualize consensus results using dot or bar plots, which are fully customizable in real time. Parameters such as color gradients, axis labels, number of terms shown, and text sizes can be adjusted instantly, allowing users to export publication-quality figures directly from the browser.

For step-by-step tutorials, practical examples, and detailed information covering all aspects of the tool, users can consult the User Guide module of the web platform.

## Results

To illustrate the utility of MetaOmixTools, we applied 2 case studies to demonstrate the critical functions of the MetaRank and MetaEnrich modules.

### Case study 1: MetaRank module: Obtaining consensus metarankings from differential expression analysis results

To demonstrate the capability of MetaRank to integrate heterogeneous gene lists, we downloaded publicly available differential expression results reported in the Meta-SCI app (https://metasci-cbl.shinyapps.io/metaSCI/) [28] derived from 4 independent GEO datasets (GSE464, GSE45006, GSE104317, and GSE133093) that compared moderate acute spinal cord injury (SCI) against control tissue. The selected datasets represent outputs from distinct experimental platforms, including the Affymetrix microarray (Rat Genome U34 Array and Rat Genome 230 2.0 Array) and the Illumina NextSeq 500 RNA sequencing platform; this diversity provides a representative sample of heterogeneous transcriptomic sources suitable for meta-analysis. Overall, we aimed to establish a consensus ranking of genes consistently deregulated following acute SCI.

For each dataset, we separated genes into 2 lists according to their logFC—up-regulated (logFC > 0) and down-regulated (logFC < 0)—and then uploaded these lists into MetaRank as independent inputs. We selected the weighted metaranking option, ranking genes based on their logFC values (File S1), and computed 2 separate consensus rankings (for up-regulated and down-regulated genes) using the RankProd basic method. A minimum of 3 occurrences across datasets was required for a gene to be included in the meta-analysis, given that GSE464 contains fewer probes than the other platforms. We set the ranking direction to descending (higher values = better) for up-regulated genes and ascending (lower values = better) for down-regulated genes; meanwhile, we ignored missing genes without penalization. Finally, we performed functional enrichment analysis of the top 200 ranked genes using GO Biological Process (BP) annotations.

**Fig. 5.**
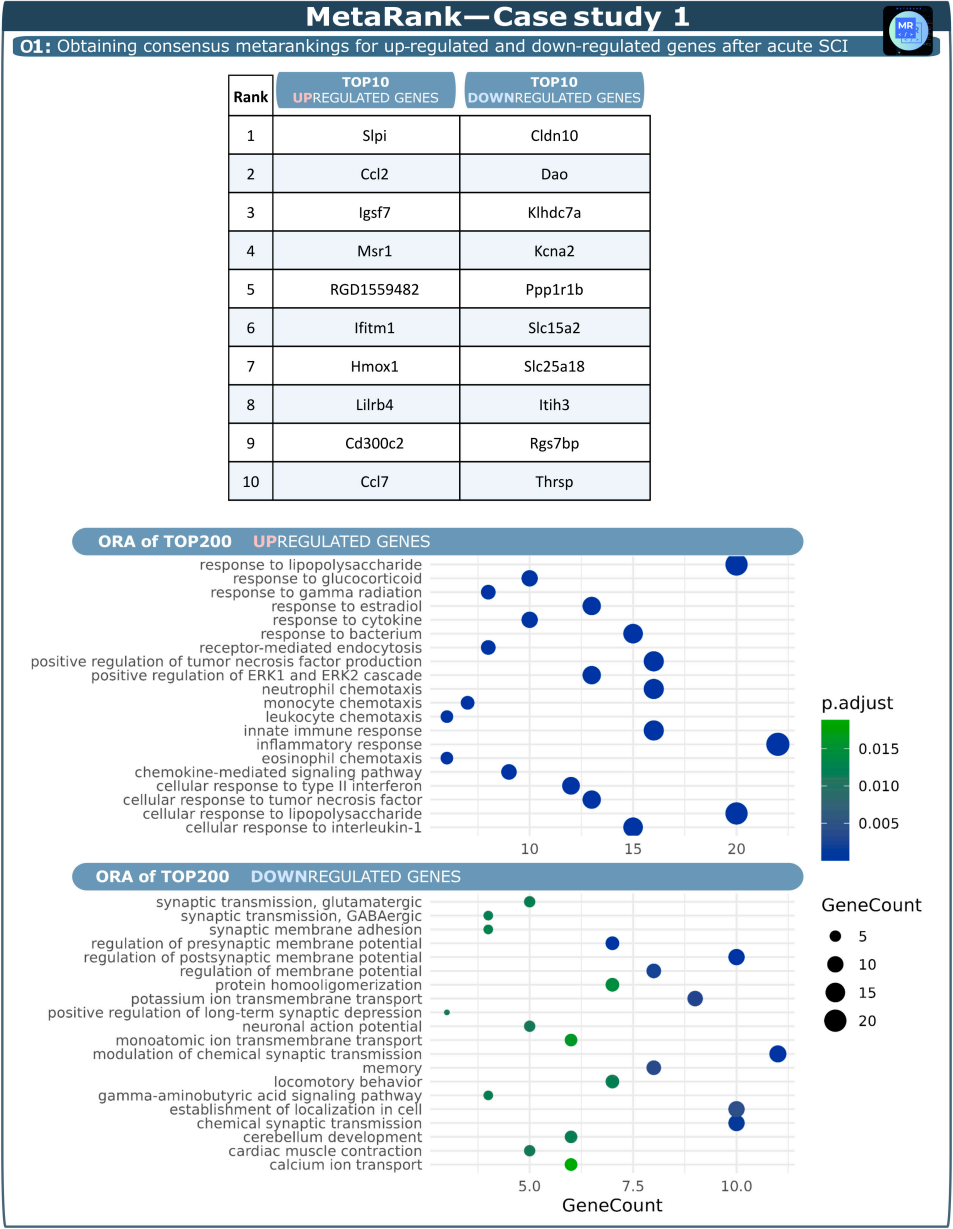
MetaRank. Case study 1. The table depicts the top 10 ranked genes for the up-regulated and down-regulated consensus lists. The associated dot plots illustrate distinct functional profiles following overrepresentation analysis of the top 200 genes in each consensus ranking.

Figure 5 displays the top 10 ranked genes for the up-regulated and down-regulated consensus lists. Among the top up-regulated genes, we found well-known inflammatory mediators, such as *Slpi* (secretory leukocyte peptidase inhibitor), *Ccl2* (C-C motif chemokine ligand 2), and *Msr1* (macrophage scavenger receptor 1), supporting their established role in immune activation after SCI [29–31]. Conversely, the top down-regulated genes included critical markers of neuronal homeostasis and excitability, such as *Kcna2* (potassium voltage-gated channel subfamily A member 2), *Ppp1r1b* (protein phosphatase 1 regulatory inhibitor subunit 1B), and *Dao* (d-amino acid oxidase), reflecting the extensive repression of neuronal signaling and loss of tissue integrity typical of the acute injury phase [32–34].

To interpret the biological significance of the consensus lists, we performed ORA on the top 200 genes from each ranking. Dot plots highlighted distinct functional profiles: we observed an enrichment of immune-related processes (Fig. 5), such as inflammatory response, chemokine-mediated signaling pathway, monocyte chemotaxis, or leukocyte chemotaxis, in the up-regulated metaranking but the marked depletion of neuronal functions, such as potassium ion transmembrane transport, synaptic transmission, glutamatergic and locomotory behavior, in the down-regulated consensus signature, which agrees with the findings reported by Grillo-Risco *et al.* [28].

This case study illustrates how MetaRank can effectively integrate multiple transcriptomic data to produce robust consensus gene rankings and highlight biologically meaningful patterns across multistudy datasets.

### Case study 2: MetaEnrich module: Identifying shared functional programs across divergent transcriptional patterns in melanoma and neurodegenerative disease

To illustrate the ability of MetaEnrich to integrate functional evidence across multiple heterogeneous gene lists, we analyzed publicly available results from the MetaFun-MBM resource (https://bioinfo.cipf.es/metafun-mbm/) [35]. Shared molecular mechanisms exist between cancer and neurodegenerative diseases, as evidenced by the secretion of Alzheimer’s-associated amyloid β by melanoma cells and the repeatedly observed correlation between melanoma and Parkinson’s disease [36–40]. To explore these connections through functional meta-analysis, we selected 4 differential expression comparisons between disease samples and their respective healthy controls: melanoma brain metastasis (MBM-3), Alzheimer’s disease in hippocampus (AD-HP), Alzheimer’s disease in cortex (AD-CT), and Parkinson’s disease in substantia nigra (PD-SN). The neurodegenerative disease signatures derive from meta-analyses [41,42], whereas MBM-3 corresponds to a single independent study [43]. Therefore, these datasets remain suitable for the MetaEnrich evaluation under realistic multistudy heterogeneity. We aimed to capture the common biological pathways affected in these diseases by analyzing functions associated with divergent gene expression patterns: genes up-regulated in cancer but down-regulated in neurodegeneration.

To obtain the gene lists of interest, we first filtered for significant genes (FDR < 0.05) in all 4 cases. We selected the top 300 most significantly up-regulated genes for melanoma and the top 300 most significantly down-regulated genes for each of the 3 neurodegenerative datasets (File S2). We uploaded the 4 gene lists as input to MetaEnrich, then performed individual ORAs for each list using GO BP as the annotation database, and applied Fisher’s method to combine *P* values, which required the evaluation of a functional term in all 4 lists before being considered for the *P*-value combination step.

**Fig. 6.**
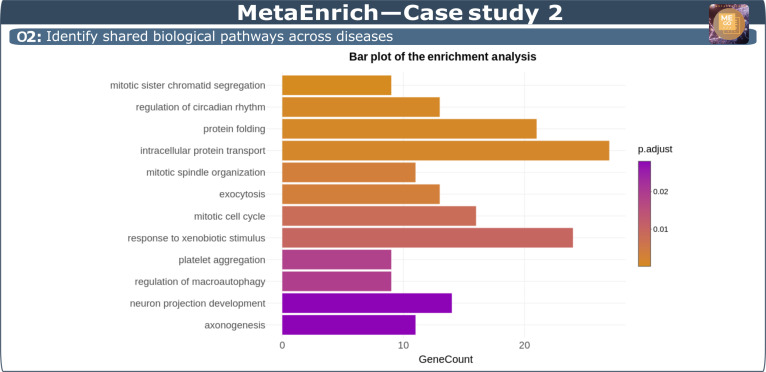
MetaEnrich. Case study 2. The bar plot displays significantly enriched biological processes identified by *P*-value combination meta-analysis.

The analysis revealed 12 significantly enriched BPs (FDR < 0.05) (Fig. 6), including processes related to cell cycle regulation (e.g., mitotic sister chromatid segregation, mitotic spindle organization, and mitotic cell cycle). The up-regulation of these processes in melanoma correlates with the cancer-associated hallmark of sustained proliferation [44]; conversely, their down-regulation in neurodegenerative diseases reflects the postmitotic nature of neurons, where aberrant cell cycle reentry often represents a prelude to neuronal apoptosis rather than division or indicates the loss of proliferative glial support [39,45,46]. Finally, we observed terms related to neuronal structural development (e.g., axonogenesis and neuron projection development). The down-regulation of these pathways in Alzheimer’s disease and Parkinson’s disease represents a direct molecular signature of neurodegeneration and synaptic loss [47,48], while their up-regulation in melanoma highlights the “neural mimicry” of these tumors. Melanoma cells, which share an embryological origin with neurons, reactivate such neural developmental programs to acquire high plasticity and invasive capabilities, facilitating metastasis to the brain [35,36].

This case study illustrates how MetaEnrich can effectively integrate gene lists of diverse origins to identify robust consensus functional terms. As demonstrated in this case by the identification of shared functional processes in melanoma and neurodegenerative diseases, the tool excels at highlighting biologically meaningful patterns from heterogeneous data sources.

## Discussion

Here, we present MetaOmixTools, a web-based suite that facilitates the widespread adoption of meta-analysis methods and makes them accessible to the broader research community. The platform integrates 2 modules, MetaRank and MetaEnrich, that allow users to generate consensus rankings and perform functional meta-analysis through *P*-value combination, respectively. In comparison with similar tools (Table S1), MetaOmixTools provides a distinct advantage, as alternative web tools usually support only one of these tasks, require raw expression matrices, or simply compare list overlaps without calculating a statistical consensus. Analyses can be carried out entirely online in an intuitive, interactive, and code-free environment, quickly producing results that can be easily explored and interpreted by experts and nonexperts alike. Additionally, it is the only platform incorporating the functionality to import and export session settings, thereby ensuring the reproducibility of the analysis.

Versatility regarding input formats and statistical strategies represents a critical advantage of MetaOmixTools over existing solutions. While other tools require users to upload strictly pre-formatted or pre-ordered lists, we designed our suite to adapt to user data. We allow researchers to paste feature lists or upload raw files in standard formats (e.g., CSV or TSV). Notably, the platform automatically sorts these inputs based on user-defined scores (e.g., logFC in ascending or descending order), saving time and substantially reducing the risk of manual formatting errors common in data preprocessing. Furthermore, the suite offers precise control over data heterogeneity; users can easily define the minimum frequency of appearance for a feature to be included in the consensus, ensuring consistency across studies. In parallel, MetaRank provides specific mechanisms to handle missing values (e.g., imputation, exclusion, or penalization), guaranteeing that the final consensus reflects biological trends rather than technical artifacts from incomplete datasets. This flexibility also extends to the statistical methodology. While comparable tools limit unweighted analysis to just 3 algorithms, MetaRank implements 6 distinct aggregation methods and, crucially, is the only platform that allows weighted list aggregation via rank product [20], filling an empty niche. Similarly, MetaEnrich outperforms platforms that rely solely on Stouffer’s *z*-score by providing a comprehensive range of *P*-value combination strategies, including the widely used Fisher’s method [21].

Although initially conceived for gene-based analyses, we designed MetaOmixTools as fully extensible to any biological feature. While the suite includes built-in functional annotations for model species (human, mouse, and rat) and widely used databases (GO, KEGG, and Reactome), users also can upload custom annotation files to perform enrichment analyses with their own feature identifiers, making the suite adaptable to a diverse range of organisms and biological elements (e.g., proteins, metabolites, or microRNAs). This flexibility expands the potential applicability of MetaOmixTools across different research contexts. Another strong point of the suite is that MetaEnrich can also work with single lists, functioning as a conventional enrichment tool alongside its main multilist meta-analysis functionality, providing users with an extra level of versatility within the same interface.

We designed MetaOmixTools with a strong emphasis on usability and publication-ready outputs. Unlike many other web tools that offer static visualizations, the suite generates interactive, highly customizable graphics (content and style), allowing users to modify colors, labels, and layouts to create publication-ready figures. Critically, we address the common lack of support in web-based tools by integrating an extensive help section, detailed tutorials, and contextual help messages throughout the whole analysis pipeline.

To address analytical reproducibility, MetaOmixTools incorporates specific tracking and session mechanisms. The application is hosted within a containerized Docker architecture, meaning that the computational environment remains stable and unaffected by external package updates. For each analysis, users can download a session parameter file that logs the exact configuration, fixed random seeds for permutation-based calculations, and version numbers of the underlying R packages and annotation databases. This file can be reloaded into the platform to automatically restore the exact interface state and parameters from a prior analysis. Additionally, the suite includes an automated report generator that compiles all parameters, interactive tables, and custom plots into a single portable HTML document.

Despite its capabilities and methodological flexibility, users must consider the inherent limitations associated with integrative approaches. MetaOmixTools relies on precomputed results generated by independent pipelines, meaning that differences in initial preprocessing, normalization, and statistical methodologies can introduce technical bias. While rank aggregation and *P*-value combination are specifically designed to mitigate this noise and extract conserved signals from highly heterogeneous sources, the validity of the downstream consensus heavily depends on the biological comparability of the inputs. Furthermore, we acknowledge the widely known limitations of ORA [49], specifically its sensitivity to arbitrary threshold selection. Therefore, we recommend testing different numbers of top features to evaluate enrichment stability and robustness of the results. The ultimate responsibility for setting inclusion criteria, data curation, and assessing dataset independence remains with the researcher, as the platform operates strictly as a statistical engine. Finally, we emphasize that *in silico* meta-analyses conducted via MetaOmixTools are fundamentally hypothesis generating; any causal relationships inferred from the consensus results must be corroborated through rigorous laboratory validation. We strongly advise users to consult the online manual for comprehensive information regarding all statistical aspects, parameter selection, and practical recommendations for both the MetaRank and MetaEnrich modules.

In summary, MetaOmixTools provides a unified, accessible solution for ranked list and functional enrichment meta-analysis. By combining flexibility, interactivity, and methodological robustness, MetaOmixTools empowers the bioinformatics community to integrate multi-omics datasets and extract meaningful biological insights in a simple and reproducible way.

## Data Availability

The code is available at https://github.com/emekate00/metaomixtools. The web tool is openly available at https://bioinfo.cipf.es/metaomixtools/.
